# Supplementary material for: Are carbon emissions trading and green financial instruments synergistic? -Comprehensive quantitative research based on content analysis
Source: PLoS One. 2024 Mar 7;19(3):e0298601. doi: 10.1371/journal.pone.0298601 (PMC10919601; doi:10.1371/journal.pone.0298601)
Supplement: S1 Data — (ZIP) [file pone.0298601.s001.zip › Data/地级市Z_F工作报告.zip!/╔╜╢1⁄2/║╩╘≤╩╨/└┤╫╘╣1⁄2╓┌║┼í╛┬φ┐╦╩2╛▌═°í┐_2014.docx]

各位代表:

　　现在，我代表市人民政府向大会报告工作，请予审议，并请市政协各位委员提出意见。

　　一、2013年工作回顾

　　去年以来，面对国际环境错综复杂、国内经济下行压力较大的严峻形势，全市人民在市委的坚强领导下，深入落实科学发展观，大力实施“双轮驱动”战略，全面加快“转调创”步伐，着力培育“五大主导产业”，切实保障和改善民生，较好地完成了经济社会发展目标任务。

　　（一）经济平稳较快增长。预计实现地区生产总值2052亿元，同比增长12%；完成公共财政预算收入159.3亿元，按可比口径增长6.3%；城镇居民人均可支配收入21150元、农民人均纯收入9130元，分别增长10.5%、11.5%；完成固定资产投资810亿元，增长23%；实际到位外资2.2亿美元，增长33%。引进落地过亿元项目220个，过10亿元项目53个。在建亿元以上项目455个，100个市重点项目超额完成投资计划。规划培育“五大主导产业”，能源化工、生物医药、机电设备制造、农副产品加工实现主营业务收入4945亿元，增长19%；商贸物流业交易额突破2000亿元，增长14%。民营经济加快发展，新注册私营企业4523家、个体工商户26327户，分别增长21.7%、11.6%。金融机构存款余额1917.7亿元、贷款余额1230.7亿元，分别比年初增加309.7亿元、168.3亿元。

　　（二）质量效益稳步提升。预计三次产业比调整为12.4:54.6:33，二三产业比重提高1.1个百分点。加快推进现代农业发展，粮食总产134.6亿斤，实现“十连增”，为全省粮食增产做出了重要贡献；土地流转面积达到104.4万亩；标准化基地发展到320万亩；牡丹产业化走在全国前列，种植面积达到46万亩；农作物耕种收综合机械化率提高到83%；规模以上农业龙头企业增加到1405家，农民专业合作社发展到9857个，带动农户50余万户。精心组织工业运行，规模以上企业新增202家、达到2607家，预计实现主营业务收入5450亿元、利税720亿元，分别增长18.2%、17%；入库税金过亿元的企业达到10家，主营业务收入过50亿元的达到7家。加大服务业扶持力度，预计完成服务业增加值677亿元，增长12%；社会消费品零售总额突破千亿元，增长13.3%。积极推动外经贸发展，预计完成进出口总额32亿美元。扎实推进金融工作，新增各类金融机构14家、区域性股权交易市场挂牌企业4家，浦发银行筹建完毕。着力提高财政收入质量，税收占财政收入的比重提高到77.6%、超过全省平均水平；主体税种占税收的比重达到57%，按可比口径提高5.9个百分点。

　　（三）城镇建设扎实推进。预计城镇化率达到41.6%，提高1.6个百分点。县城总体规划修编和30个重点镇总体规划全部完成，8个项目获省优秀城市规划设计奖。突出抓好中心城市建设，启动实施城建工程186项，完成政府性投资10.2亿元，市图书馆开馆试运行，规划展览馆、文化艺术馆启动建设，打通中和路、振兴路等断头路5条，新建改造长城路、仓南路等道路25条，铺设雨污管网76公里，新增绿化面积717万平方米，国家园林城市创建通过专家组评审。加快县城和镇村建设，县城新增建成区面积12平方公里、人口11万，新增省级示范镇11个，建成农村新型社区270个。积极推进城市综合开发，房地产市场平稳发展。大力推进重大基础设施建设，菏泽机场纳入国家民航发展规划并启动前期工作，菏泽火车站完成站场改造、新站房加快施工，10座平原水库开工建设，德商高速、东新高速建设和济董路、砀定路升级改造进展顺利，220国道竣工通车。

　　（四）转方式取得积极进展。深入实施创新驱动，完成全社会研发投入21亿元，占地区生产总值的比重突破1%；新增省级企业技术中心7家、工程研究中心6家、创新型企业9家、中国驰名商标1件、山东省著名商标14件；国家“千人计划”、博士后科研工作站和省级科技孵化器建设均实现零的突破；预计完成高新技术产业产值1600亿元，占规模以上工业的30%，达到全省平均水平；我市首次被评为“全国科技进步先进市”。深入推进节能降耗，淘汰高耗能落后设备1200台（套），累计节能32万吨标准煤。坚持节约集约用地，挖潜盘活存量土地1.2万亩，新增耕地5.1万亩。扎实开展大气、水污染防治专项行动，空气质量逐步改善，三条主要出境河流水质稳定达标，代表山东再夺迎淮核查第一名。

　　（五）民生状况明显改善。民生支出占公共财政预算支出的比重达到67.6%。圆满完成承诺的33件民生实事，增加实施了打通城区断头路、建设丹阳立交桥等6件民生实事。新增城镇就业10.1万人、农村劳动力转移就业24.5万人、社会保险参保18.1万人；城乡医疗救助标准均提高500元，城市低保月保障标准和农村低保年保障标准分别提高70元、200元，农村五保集中、分散供养标准分别提高1300元、900元；开工建设中心敬老院34处、建成投用17处，社会养老体系建设全面展开。公开招录教师2400名，新改建中小学校舍107万平方米，新建幼儿园262所、城区中小学12所，齐鲁工业大学菏泽校区加快筹建。第一批医改试点进展顺利，市立医院完成改扩建工程，公办基层医疗卫生机构全部实行药品零差率销售，新农合政府补助标准提高40元，20种重大疾病纳入医疗保险，10项基本公共卫生服务项目深入实施，“先诊疗、后付费”模式全面推行。开工保障性住房1.15万套，改造农村危房1.72万户；新增农村饮水安全人口62万人，改造农村公路1658公里、窄危桥41座。新增集中供热覆盖面积320万平方米、民用天然气用户3.5万户；深入开展交通秩序专项整治，新增公交线路6条、新能源公交车110辆。县区“两馆”主体基本完工，新建农村文化大院500个、村级健身工程856处，新增有线数字电视用户51万户，送戏下乡1382场、送电影下乡7万场；圆满完成“十艺节”参赛办赛任务，9个剧目获国家级奖项。积极排查化解矛盾纠纷，加强社会治安综合治理，不断强化安全生产和食品药品质量监管，社会大局保持稳定。同时，人口计生、妇女儿童、邮政通讯、气象、人防、统计等事业持续发展，民族宗教、外事侨务、人民武装、防震减灾、物价、对台、史志、档案等工作取得新的成绩。

　　（六）政府自身建设不断加强。深入开展“作风改进年”、治理“庸懒散”、优化营商环境等活动，精简行政审批事项127项，压缩办理时限64%，市级中介组织与行业主管部门实现分离，涉企收费标准大幅降低，服务效能明显提升。修订了县区科学发展观摩、考核办法，完善政务督查联动机制，出台鼓励“飞地经济”、促进园区建设、支持生物医药产业发展、提高外经贸水平等多项政策措施，促进了工作落实和发展方式转变。理顺了高新区管理体制，整合了食品药品安全监管职能，财税、文化、医疗卫生、社会保障等重点领域改革取得积极进展。自觉接受人大法律监督、工作监督和政协民主监督，认真办理人大代表议案、建议和政协委员提案，办复率100%。严格落实党风廉政建设责任制，强化行政监察和审计监督，政府系统廉政建设得到加强。

取得上述成绩，实属不易，凝聚了全市上下、方方面面的心血和汗水。在此，我代表市人民政府，向全市人民，向全体人大代表、政协委员，向各民主党派、工商联、人民团体和社会各界人士，向离退休老领导、老同志，向驻菏部队、武警官兵和中央、省驻菏单位，向所有关心支持菏泽发展的同志们、朋友们，表示崇高的敬意和衷心的感谢！

　　我们也清醒地认识到，工作发展中仍然存在不少困难和问题。主要是：欠发达的市情没有改变，加快发展的任务依然艰巨；发展的质量效益不高，投入产出率低，资源消耗高，创新能力弱，产业竞争力不强；资源环境约束加剧，土地集约利用水平较低，大气和水污染防治任务繁重；地方财力薄弱与改善民生刚性需求的矛盾比较突出，一些群众期盼的问题尚未得到很好解决；政府破解发展难题的能力、依法行政水平和工作效率有待进一步提升等。对此，我们一定高度重视，采取有力措施，认真加以解决。

　　二、2014年目标任务

　　今年是深入贯彻党的十八届三中全会精神的开局之年，是全面落实习近平总书记视察菏泽指示精神的重要一年，也是完成“十二五”规划任务的关键一年。工作中必须牢牢把握“稳中求进、改革创新”的核心原则，立足我市欠发达实际，以全面深化改革为动力，以提高发展质量和效益为中心，以招商引资和项目建设为抓手，大力实施“双轮驱动”战略，全力加快“转调创”步伐，着力培植“五大主导产业”，不断加强生态文明建设，切实保障和改善民生，努力推动经济社会科学发展、加快发展。

　　综合考虑多方面的因素，建议今年经济社会发展的主要预期目标确定为：地区生产总值增长10%；公共财政预算收入增长7%；规模以上工业增加值增长15%；规模以上固定资产投资增长16%；社会消费品零售总额增长12%；进出口总额增长15%；城镇居民人均可支配收入增长10%，争取农民人均纯收入增长更高一些；登记失业率控制在4%以内；人口自然增长率控制在9.3‰以内；节能减排完成省下达的任务。

　　上述指标比往年有所调低，主要原因有三点。一是与中央和省实现工作指导转变的要求相衔接。中央和省经济工作会议都明确指出，不再简单以地区生产总值增长率论英雄，把推动经济发展的立足点转到提高质量和效益上来，并调低了增长指标。二是符合菏泽现阶段发展特征。经过近年来的快速发展，我市进入适度调整阶段，调低增长指标符合发展规律，切合菏泽实际。三是有利于实现更高水平的发展。调低增长指标，能为深化改革和转方式调结构留出空间，有利于引导各级追求实实在在的发展，追求干部群众真正得实惠的发展，追求质量和效益得到提高而又不会带来后遗症的发展。

　　三、坚持深化改革，全面激发发展活力

　　改革是时代的最强音，更是发展的源动力，必须按照中央和省、市委要求，扎实推进各项改革，破除体制机制障碍。一是围绕转变政府职能深化改革。进一步简政放权，再取消下放一批行政审批事项。推进行业协会、中介组织与行政机构脱钩，努力打破“官垄断”。健全优化营商环境长效机制，继续推进工商注册便利化，巩固扩大“三项重点工作”成果。加大政府向社会购买服务力度。二是围绕激发市场活力深化改革。深入实施国有企业股权多元化改革，推进国有企业发展为混合所有制经济。健全技术创新市场导向机制。加快农村信用社银行化改革步伐。三是围绕活跃农村经济深化改革。全面推进农村集体土地所有权、家庭承包经营权、宅基地使用权和建设用地使用权确权登记发证工作，加快建设农村产权流转交易平台，积极构建立体式复合型现代农业经营体系。鼓励引导工商资本到农村发展适合企业化经营的现代种养业，向农业输入现代生产要素和经营模式。四是围绕推进城乡一体化深化改革。理顺市区综合开发工作机制，调动牡丹区、开发区、高新区的积极性。完善土地收储制度，创新城建融资机制，吸收社会资本参与，做大政府融资平台，加快推进棚户区和城中村改造。放开城市落户限制，搞好各项社会保障衔接，促进进城农民有序市民化。五是围绕保障改善民生深化改革。探索实施校长职级制和县域内教师定期轮岗交流制度，鼓励支持社会力量办学，促进城乡教育均衡发展。积极推进养老保险、医疗保险整合，加大救助资源统筹力度，推进城乡低保统筹发展。加快推动政府由办文化向管文化转变。启动第二轮县级公立医院综合改革试点，鼓励引导民营医院加快发展。做好计划生育政策调整的衔接工作，促进人口均衡发展。六是围绕规范财税管理深化改革。完善征收机制，堵塞“跑冒滴漏”，杜绝虚收空转，优化财政结构。规范财政支出，推行“全口径”预算，完善“花钱必问效，无效必问责”制度。健全国有资产运营监管机制，规范公共资源交易，确保国有资产保值增值。七是围绕创新社会治理深化改革。加快网格化管理步伐，深化村（居）务公开，完善安全隐患排查治理和预防控制体系，建立更严格的食品药品监管制度，进一步健全矛盾纠纷排查化解和突发事件应急救援机制，不断提高群众安全感和满意度。

　　四、坚持转型升级，推动经济科学发展、加快发展

　　当前，欠发达仍然是菏泽最大的实际，加快经济发展仍然是菏泽最大的政治、最硬的道理和最根本的任务，必须紧紧抓住质量和效益这个中心，全力加快发展步伐。

　　（一）以更高的境界推进新型工业化。菏泽发展落后，突出表现为工业总量小、发展层次低，要摆脱欠发达面貌，必须下大力膨胀工业规模、提升工业档次。一是努力提高招商引资和项目建设水平。把招商引资作为扩大投入的主渠道，大力转变招商方式，坚决摒弃“捡到篮子都是菜”的做法，坚决不上高耗能、高污染、低效益、产能过剩的项目；坚持“铺天盖地”与“顶天立地”并重，全力引进产业配套能力强、科技含量高、财政贡献大的项目，不断提高投入产出比。高水平搞好项目策划，确保纳入国家和省计划的项目个数居全省前列。完善领导干部分包重点项目机制，推动100个市重点项目早开工、早投产、早达效。认真落实加快11个省级园区发展的意见，强化基础设施配套，完善商住、物流等服务体系，促进项目向园区聚集。二是着力培育壮大主导产业。以打造终端产品为目标，围绕补齐完善产业链条和打造产业集群，着力引进具有核心竞争力和较强配套拉动作用的项目，倾力打造能源化工、生物医药、机电设备制造、农副产品加工四大产业。推动关联企业协同协作，大力发展绿色低碳循环经济，促进高端高质高效发展，切实增强四大产业的综合竞争力。三是努力做优做强现有企业。突出抓好骨干企业培育，加大扶持力度，强化科技创新，促进内涵发展，努力把百强企业打造成全市现代管理的标兵、科技创新的先锋、奉献社会的楷模、经济发展的支撑。加大低效企业排查力度，帮助其分析存在问题、解除瓶颈制约，最大限度地释放闲置产能；对停产、半停产而又无法盘活的企业，以及占用大量土地、长期没有贡献的企业，坚决“腾笼换鸟”。强化企业家培训，使他们提升境界、拓展思路，搞好长远规划，谋求更高水平发展。四是加快推进科技创新。足额落实并充分用好科技创新专项资金，引导全社会加大投入，确保研发投入占地区生产总值的比重达到1.1%。抓好公共创新平台建设，推动开发区、高新区尽快建成国家级标准的科技孵化器，各县区加快建设省级标准以上的科技孵化器。加大高端人才引进培养力度，促进科研投入成果化、科研成果产业化，确保高新技术产业产值占规模以上工业的比重达到31%以上。五是积极发展开放型经济。加快青岛保税港区菏泽功能区建设，加强出口基地和示范区建设，落实好出口退税和进口设备增值税抵扣政策，调整优化出口结构，扩大紧缺资源、核心技术、关键装备进口，力争实现进出口总额36.8亿美元、实际到位外资2.5亿美元。

　　（二）以更高的标准推进新型城镇化。围绕构建中心城市、县城、小城镇、农村社区“四位一体”的新型城镇体系，大力推进以人为核心的城镇化，确保城镇化率提高1.5个百分点。一是提升城镇功能。按照“产城融合、设施配套、突出特色、宜商宜居”的要求，完善市县城镇发展规划，搞好功能区划分，优化城市布局。加快完善城镇水、电、路、暖、气、通讯等公共基础设施，配套建设学校、医院、市场、公交、绿地、停车场等公共服务设施，增强城镇的整体功能。抓好17个省级示范镇和30个市级重点镇建设，加快打造一批小卫星城。尊重群众意愿，稳妥推进农村新型社区建设。二是提高综合开发水平。注重开发的整体性和协调性，大力引进业界强企名企，有序推进大片区组团开发，坚决改变零打碎敲的开发模式。着力打造精品工程，条件不成熟，宁可不建、缓建，也不能仓促上马、留下遗憾。保护好菏泽城的“外圆内方”结构，以及护城河、护城堤等古迹，突出历史文化特色，让人们望得见林海、看得见水邑、闻得到花香、记得住乡愁。三是提高管理水平。加快构建市县互联的“数字城管”新模式，努力实现城镇管理全天候、全方位、全覆盖，不断提高城市精细化管理水平。着力加强老城区、背街巷等重点区域和城市公共卫生、绿化、亮化等重点领域管理，搞好城乡环境综合整治，下大力解决脏乱差、管理不规范等问题，进一步巩固园林城、文明城、卫生城创建成果。四是加快重大基础设施建设。强化组织领导，克服一切困难，全力推动东新高速、济祁高速、砀定线西延等交通重点工程，确保德商高速菏泽段建成通车、菏泽火车站全面竣工运营，菏泽机场完成前期工作、具备开工条件；竭力推进曲阜东经菏泽至兰考铁路客运专线前期工作；加快13座平原水库、坚强智能电网、洙水河航道等工程建设，为长远发展打下坚实基础。

　　（三）以更高的层次推进农业现代化。充分发挥农业大市优势，按照规模化种养、标准化生产、品牌化销售、产业化经营的要求，加快推进农业由大向强转变。一是抓好农产品基地建设。完善农产品质量安全保障体系，加强农田水利基本建设，大力发展无公害、绿色、有机农业。在稳定粮食生产、继续为国家粮食安全做贡献的同时，重点抓好牡丹、芦笋、山药、中药材等特色农作物的规模种植。力争新发展标准化生产基地50万亩、牡丹25万亩。加快发展林下养殖，新增标准化养殖示范场区110个。抢抓习近平总书记视察牡丹产业的机遇，强化扶持推动，探索种植保险，加大研发、营销力度，拉伸产业链条，力争早日把牡丹产业打造成特色支柱产业。二是推进农业产业化经营。积极培育新型经营主体，促进土地向专业大户、家庭农场、农民合作社和农业企业流转，大力发展多种形式的规模经营。按照“龙头带基地、基地连农户”的模式，加快打造特色加工集群。确保新增市级以上示范合作社100家、市级龙头企业20家，农副产品加工业实现主营业务收入2250亿元。三是强化农业科技支撑。突出抓好农业科技人才队伍建设、新型职业农民培训和农业新品种、新技术、新成果的引进推广，促进农业技术集成化、劳动过程机械化、生产经营信息化，努力为农业插上科技的翅膀。确保全年培训农民20万人次，科技对农业增长的贡献率达到54%以上。

　　（四）以更快的速度推进服务业发展。发展服务业是调结构、稳增长、促就业、富民富财政的重大举措，必须按照提质增速的要求，全力推动、加快发展，确保占地区生产总值的比重提高1个百分点。一是突出发展商贸物流业。按照“一核两轴四区多中心”的总体布局，围绕重要资源、重点产业和货物集散等，加快建设大型商贸物流园区、专业批发市场、骨干商贸物流企业和配套支撑项目，积极发展电子商务、连锁经营、物流配送等现代经营方式，确保商贸物流业交易额增长14%以上。二是大力发展文化旅游业。强化文化和旅游的深度融合，推进重点景区和配套设施建设，搞好旅游产品开发、旅游线路策划，举办好国际牡丹文化旅游节，积极发展乡村旅游，加快打造“牡丹之都、好汉之乡”旅游品牌，确保旅游总收入增长15%以上。三是加快发展金融业。大力引进各类金融机构，积极推进企业上市，规范民间融资管理，确保新增社会信用总量340亿元。四是积极发展新兴服务业。充分发挥林交会、农资会、住博会等展会的品牌效应，推动会展业向高层次、常态化发展；积极发展服务外包、科技服务、物联网、邮政、通讯、信息、创意等新兴业态，不断培育新的经济增长点。

　　（五）以更大的力度推动县域经济特色发展。按照习近平总书记“因地制宜、特色发展”的指示要求，引导各县区大力发展特色经济，努力实现差异竞争、错位发展。一是促进“三区”高端发展。按照高端高效、绿色低碳、宜居宜业的要求，引导开发区、高新区、牡丹区加快转变发展思路，努力在全市转型发展中争当领头雁、排头兵。支持开发区大力发展外向型经济，加快改造提升现有产业，力争早日晋升为国家级开发区。支持高新区倾力打造“高新”品牌，大力发展新医药、新材料、新信息产业，加快创建国家级高新区。鼓励牡丹区发挥中心城区优势，做好“退二进三”文章，突出抓好城市综合开发、现代服务业发展和牡丹产业培育，努力实现“凤凰涅槃、浴火重生”。 二是推动各县协调发展。鼓励先进县率先突破，支持定陶、鄄城、成武加快赶超。引导各县立足资源禀赋、区位条件和产业基础，重点培植2-3个主导产业，努力形成各具特色的产业体系。认真落实“飞地经济”发展政策，推动项目按产业定位跨县域、跨园区布局，避免低水平重复、同质化发展。三是推进乡镇加快发展。把壮大镇域经济作为扩大就业、富裕农民、支撑县域发展的重要战略，推动各乡镇按照“一镇一业，一村一品”的要求，依托资源优势和现有基础，大力发展板材、果蔬、纺织、草柳编等特色加工，不断提升加工档次，倾力打造特色板块经济，努力形成中小微企业“铺天盖地”、竞相发展的强劲态势。出台促进民营经济发展政策，降低准入门槛，最大限度地调动民资、集中民智、激发民力，努力营造“千军万马”上项目、闯市场、办企业的浓厚氛围，不断为县域经济注入新的活力。

　　五、坚持生态优先，大力加强生态文明建设

　　牢固树立“绿水青山”就是“金山银山”的发展理念，以“壮士断腕”的勇气，狠抓生态治理和环境保护，坚决守护好菏泽这片蓝天、碧水、净土。一是加大水污染防治力度。强化项目审批管理，严格落实“三同时”制度，坚决把高污染、高能耗项目拒之门外，决不要变了色、带了味的GDP。完善“治、用、保、防、控”流域治污体系，加强农村面源污染治理，进一步落实“河长制”，全面实施涉水企业“十个一”工程，加快污水、垃圾处理设施建设，力争市区、县城建成区管网铺设到位、污水和垃圾收集处理到位，确保河流水质持续改善。二是狠抓大气污染防治。完善属地管理和行业管理双重责任制，全面加强建设工地、拆迁现场、渣土运输、道路保洁等重点领域管理，推进集中供热工程建设，加快燃煤锅炉、“黄标车”淘汰步伐，抓好重点行业脱硝脱硫、油烟净化和工业异味治理，促进空气质量逐步改善、“蓝天白云”天数不断增加。三是强力推进节能降耗。严格落实节能评估审查、新增能耗等量淘汰制度，坚决执行淘汰落后产能清单计划，扎实开展企业能效对标活动，全面实施节能技改工程；巩固扩大可再生能源建筑应用示范城市创建成果，统筹推进建筑、交通、公共机构等各个领域节能，确保节能35万吨标准煤。四是全面促进资源集约节约利用。严格资源利用许可制度，大力推广节水节材新技术，突出抓好郓城煤化工园区和30个循环经济重点项目建设，努力降低资源消耗。加强水源地保护和用水总量管理，加快建设节水型社会。严格规范土地使用管理，坚决遏制违法违规用地行为；加大低效闲置土地整治力度，鼓励支持企业“零增地”技改，提高土地集约节约利用水平。五是抓好生态环境建设。深入开展植树造林和城区裸露土地绿化，抓好河道生态修复和湿地公园建设，加强采煤塌陷区综合治理，搞好生态文明乡村建设，推进乡村文明行动，促进城乡环境持续改善。

　　六、坚持以人为本，着力保障改善民生

　　坚持民生优先不动摇，把“民之所望”作为“政之所向”，持续加大民生投入，让发展成果更多更公平地惠及全市人民。今年重点办好7个方面30件民生实事。城乡就业方面：强化就业培训，完成技能培训7万人；抓好重点群体就业，新增城镇就业9万人、农村劳动力转移就业18万人，帮扶困难群体就业4500人；加强创业带动就业，新增创业7500人，带动就业2万人。社会保障方面：农村低保年保障标准提高300元；医疗救助最高限额提高5000元；城乡医保财政补助标准提高40元；新建中心敬老院16处，每千名老人拥有社会养老床位数达到26张；为60周岁以上老年人每年免费查体一次，为90周岁以上老年人发放高龄补贴；开工保障性住房1万套以上，改造棚户区6600户、农村危房5000户。教育发展方面：新、改扩建农村义务教育学校校舍120万平方米；新建城区学校14所以上，新建市级高中一处，完成菏泽一中南校区扩建和菏泽一中老校区、市实验小学北校区改建；新建幼儿园200所；创建省级规范化职教中心2处；齐鲁工业大学菏泽校区建设取得明显进展；公开招录中小学、幼儿园教师2000名以上；救助城乡低保和计划生育贫困家庭大学新生各1000名，确保不让一名学生因家庭贫困而失学。医疗服务方面：免费为城乡居民提供11项基本公共卫生服务；加快市直6家医疗卫生机构扩建步伐，新增建筑面积15万平方米；推动曹县、鄄城、东明等县级医院迁建加快实施。改善居民生活条件方面：新增集中供热覆盖面积300万平方米；新增民用天然气用户5.5万户，其中市区2万户；新增农村饮水安全人口80万人；市区增加新能源公交车100辆。文化体育方面：新建农村文化大院350家、文化广场200处；新建数字化农家书屋200家，充实提升农家书屋500家；完成送戏下乡1450场、送电影下乡7万场；规划市体育中心；开工建设市体校新校区；完成50个乡镇、800个村级农民健身工程。扶贫开发方面：完善扶贫方案，强化帮扶措施，确保30万人脱贫。

　　七、坚持从严治政，切实加强自身建设

　　打铁必须自身硬。围绕完成任务目标，我们一定按照“为民、务实、清廉”的要求，深入开展“作风提升年”活动，大力加强自身建设，努力提高工作效率和服务水平。

　　（一）打造责任政府。把是否忠诚履职、尽心尽责，作为检验政治品格的重要尺度，把为全市人民谋福祉作为人生的最大追求，做到在其位、谋其政、尽其职。大力弘扬“勇于担当”精神，只要有利于菏泽发展，就毫不动摇坚持、义无反顾推动，决不见了问题躲着走、遇到困难绕着走，决不当随风摇摆的“墙头草”和“老好人”。大力倡导“功成不必在我”的境界，甘当改革发展路上的铺路石，“不贪一时之功，不图一时之名”，多做打基础利长远的工作，多做功在当今利在后世的实事好事。在发展中，着力争取有质量有效益的速度，着力争取能真正让干部群众得实惠的速度，坚决不做杀鸡取卵、竭泽而渔、寅吃卯粮的事情，坚决不让今天的政绩成为明天的包袱。

　　（二）打造务实政府。扎实开展党的群众路线教育实践活动，聚焦“四风”抓整改，重拳整治“庸懒散”，下决心解决“中梗阻”、“堰塞湖”等突出问题。牢固树立宗旨意识，自觉深入群众，密切联系群众，主动到问题多、困难大的地方倾听民声、为民解难。大力弘扬“滴水穿石”精神，咬定目标不放松，一张蓝图绘到底，一个号子喊到底，一年接着一年干，以“抓铁有痕，踏石留印”的作风，全力推动工作落实。健全督查联动机制，加大考核奖惩力度，说实话、办实事、求实效，坚决不搞华而不实的形式主义、劳民伤财的政绩工程、欺世盗名的浮夸之风。

　　（三）打造法治政府。把依法行政贯穿于工作的各个环节，自觉接受人大的法律监督、工作监督和政协的民主监督，高度重视新闻媒体和社会公众监督，支持审判、检察机关依法独立行使职权。深入推进科学民主决策，凡与群众利益密切相关的重大决策，一律坚持公众参与、专家论证、风险评估、合法性审查。严格按法定权限和程序履行职责，严厉查处有令不行、有禁不止和失职渎职行为，坚决维护法律法规的尊严。

　　（四）打造廉洁政府。牢固树立过紧日子的思想，在任何时候、任何情况下，艰苦奋斗的传统决不丢，勤俭节约的要求永不忘。始终绷紧廉洁自律这根弦，自觉做到堂堂正正做人、干干净净做事、清清白白为官，维护人民公仆的良好形象。坚持反腐倡廉常抓不懈，拒腐防变警钟长鸣，严格用制度管权、管人、管事，坚决纠正损害群众利益的不正之风，严厉查处各类违法违纪案件，切实做到干部清正、政府清廉、政治清明。

各位代表，改革鼓舞人心，发展催人奋进。让我们在市委的坚强领导下，深入贯彻落实党的十八届三中全会精神，切实凝聚同心筑梦的强大合力，努力谱写富民兴菏的崭新篇章！
